# Intestinal anti-inflammatory effects of goat whey on DNBS-induced colitis in mice

**DOI:** 10.1371/journal.pone.0185382

**Published:** 2017-09-28

**Authors:** Daline F. S. Araújo, Gerlane C. B. Guerra, Maria Manuela E. Pintado, Yasmim R. F. Sousa, Francesca Algieri, Alba Rodriguez-Nogales, Raimundo F. Araújo, Julio Gálvez, Rita de Cássia R. E. Queiroga, Maria Elena Rodriguez-Cabezas

**Affiliations:** 1 Faculty of Health Sciences of Trairi, Federal University of Rio Grande do Norte, Santa Cruz, Brazil; 2 Department of Biophysics and Pharmacology, Biosciences Center, Federal University of Rio Grande do Norte, Natal, Brazil; 3 School of Biotechnology, Catholic University of Portugal, Porto, Portugal; 4 Technology Center, Federal University of Paraíba, João Pessoa, Brazil; 5 CIBER-EHD, Department of Pharmacology, ibs.GRANADA, Center for Biomedical Research (CIBM), University of Granada, Granada, Spain; 6 Department of Morphology, Histology and Basic Pathology Biosciences Center, Federal University of Rio Grande do Norte, Natal, Brazil; 7 Department of Nutrition, Health Sciences Center, Federal University of Paraíba, João Pessoa, Brazil; "INSERM", FRANCE

## Abstract

This study evaluated the intestinal anti-inflammatory effects of goat whey in a mouse model of colitis induced by 2,4-dinitrobenzenesulfonic acid that resembles human IBD. At a concentration of 4 g/kg/day, the goat whey improved the symptoms of intestinal inflammation, namely by decreasing the disease activity index, colonic weight/length, and leukocyte infiltration. Moreover, goat whey inhibited NF-κB p65 and p38 MAPK signaling pathways and consequently down-regulated the gene expression of various proinflammatory markers such as IL-1β, IL-6, IL-17, TNF-α, iNOS, MMP-9, ICAM-1. Also, goat whey increased the expression of proteins such as mucins, occludin proteins and cytokine signalling suppressors. The immunomodulatory properties of goat whey were also evaluated *in vitro* using the murine macrophage cell line Raw 264 and CMT-93 cells derived from mouse rectum carcinomas. The results revealed the ability of goat whey to inhibit the production of NO and reduce IL-6 production in LPS-stimulated cells. In conclusion, goat whey exhibited anti-inflammatory effects in the DNBS model of intestinal inflammation, and these observations were confirmed by its immunomodulatory properties in vitro. Together, our results indicate that goat whey could have applications for the treatment of IBD.

## Introduction

Inflammatory bowel disease (IBD) is a group of chronic inflammatory bowel conditions, including Crohn's disease and ulcerative colitis, which are caused by an excessive immune system response. The most important symptom is chronic inflammation of the gastrointestinal tract with periods of exacerbation followed by intervals of remission [1,2]. IBD is associated with an increase in pro-inflammatory cytokines such as interleukin (IL)-1β, IL-6, IL-17, tumour necrosis factor (TNF)-α, prostaglandins and nitric oxide (NO), which may impair colonic muscle contraction and barrier function. These inflammatory mediators are important for the generation and maintenance of inflammation [3,4].

Activation of Mitogen Activated Protein Kinases (MAPK) pathway has been implicated in the pathogenesis of IBD. Activated MAPKs can bind and stimulate other kinase targets, translocate to the nucleus and activate transcription of pro-inflammatory genes. One of the well-studied components of MAPK signaling pathway is the nuclear transcription factor kappa B (NF-kB), which strongly influences the course of mucosal inflammation [5,6]. Activation of NF-κB can be initiated by different stimuli including bacterial cell wall components such as lipopolysaccharides, pro-inflammatory cytokines, viruses and DNA damaging agents [7].

The current pharmacological treatments for IBD include 5-aminosalicylic acid, azathioprine, mercaptopurines, cyclosporine and corticosteroids [8,9]. The introduction of biological treatments such as monoclonal anti-TNF-α antibodies, which have demonstrated clinical efficacy, represents a major recent advance in IBD therapy, but these treatments also increase the risk of infection and hypersensitivity [10,11]. Although these pharmacological innovations have improved the quality of life of patients, the induction of remission of the disease is accompanied by side effects caused by the drugs [12,13].

As an alternative or a complement to pharmacological treatments, the effects of functional foods have been extensively studied, and some contain compounds that possess intestinal anti-inflammatory activity. In this sense, goat milk and its derivatives are promising functional foods for health promotion and disease prevention. They contain bioactive components such as peptides, fatty-conjugated linoleic acid (CLA) and oligosaccharides, among others. Whey proteins and bioactive peptides that are released by hydrolysis may have beneficial biological effects on the body, such as antioxidant and anti-hypertensive effects [14].

There has been increasing interest over the last decade in CLA, which is mainly found in dairy products from ruminants. Numerous health benefits have been attributed to CLA such as anticarcinogenic, anti-atherogenic, antidiabetic, anti-inflammatory, antioxidant and immunomodulatory effects based on studies involving cell lines, animal experimental models [15–17] and modulation of the immune response in patients with Crohn's disease [18]. Furthermore, 500 mg/kg/d of goat milk oligosaccharides [19,20] shows promise for reducing intestinal inflammation.

In fact, goat milk oligosaccharides have been shown to exhibit significant intestinal anti-inflammatory effects in experimental models of mouse colitis [19,20]. Recently, we published a study showing that the oral administration of goat milk and goat yogurt before and after the induction of colitis by acetic acid ameliorated intestinal inflammation in rats [21].

Thus, the aim of this study was to assess the effects of goat whey on intestinal inflammation induced by 2,4-dinitrobenzenesulfonic acid (DNBS) in mice and the cellular responses in the Raw 264 and CMT-93 cell lines.

## Materials and methods

### Ethics

This study was conducted in accordance with the Guide for the Care and Use of Laboratory Animals (NIH Publication No: 85–23, revised 1985), and the protocol was approved by the Ethics Committee on Animal Experimentation of the University of Granada (Spain) (Ref. No. EAEC: 2010–286).

### Collection and characterization of goat whey

The milk was obtained from crossbred Pardo-Alpine goats over approximately 50 (±10) days of lactation. The animal diet followed the recommendations of the NRC (2007) and met the nutritional requirements for lactating goats.

The milk was collected at the Experimental Unit of São João do Cariri—PB belonging to the Federal University of Paraíba (UFPB, Brazil). The cheese curd used to produce the goat whey (GW) was prepared in accordance with the protocol developed by Oliveira, Garcia, Queiroga, and Souza (2012) [22]. The GW was dried using a Buchi Mini Spray Dryer B290 (Büchi Corporation, New Castle, DE, USA).

The following tests were performed in order to characterize the GW: fat was assessed using a Gerber's butyrometer, and protein was assessed by the micro-Kjeldahl method according to the recommendations of the Association of Official Analytical Chemistry (2005). Lactose levels were assessed using a High Performance Liquid Chromatograph (VARIAN, Waters 26 2690, California, USA) with a refractive index detector coupled with a Hi-Plex Ca column at 85°C using ultrapure water as the mobile phase at a flow rate of 0.6 mL/min. Fatty acids were extracted (chloroform:methanol:water—2:1:1), and fatty acid composition (including CLA) was determined by gas chromatography using an Agilent gas chromatograph, model 7890A (Agilent Technologies, Wilmington, DE, USA), coupled to a Waters Quattro micro GC model mass spectrometer (Waters Corporation, Milford, MA, USA) [23]. Lastly, the quantification of sialic acid followed the methodology used by [24].

### Reagents

All of the chemicals were purchased from Sigma-Aldrich (St. Louis, MO, USA) unless otherwise stated. The enzyme-linked immunosorbent assay (ELISA) for IL-6 and TNF-α using the mouse colonic tissue samples was conducted using the starter System® R&D (Minneapolis, MN, USA). The colonic RNA tissue was extracted with Trizol^®^ (Invitrogen Life Technologies, Life Technologies, Thermo Fisher Scientific Inc., Waltham, MA, USA). Oligo (dT) primers, Taq® DNA polymerase (Promega, Madison, WI, USA), and KAPA SYBR® FAST qPCR Master Mix (Kapa Biosystems, Wilmington, MA, USA) were used for the real-time quantitative polymerase chain reaction (RT-qPCR) analysis. The polyclonal goat antibodies against SOCs-1, iNOS, IL-17, NF-κB p65 and p38 MAPK were purchased from Santa Cruz Biotechnology (Interprise, Brazil). The streptavidin-HRP-conjugated secondary antibody and the TrekAvidin-HRP Label + Kit were purchased from Biocare Medical (Dako, USA).

### Experimental design of the colitis model

Male CD1 mice (30 ± 2.0 g) were supplied by the Laboratory Animal Service of the University of Granada (Granada, Spain) and housed in the Animal Facilities of the Center of Biomedical Investigation of the University of Granada (Granada, Spain). They were housed (6 animals per box) in standard conditions (light/dark cycle of 12 h, temperature 22 ± 0.1°C and 50–55% moisture) with free access to food (AIN-93G diet) and water.

The mice were distributed into three groups (n = 12/group): healthy, DNBS control and mice treated with GW (4 g. kg^-1^/day, dissolved in 100 μL of water). The healthy and DNBS control groups received the vehicle (water) only. GW was administered orally for 12 days, 1 hour before colitis induction and 4 days after (S1 Fig).

### The induction and assessment of colitis

On the 13th day, colonic inflammation was induced in the DNBS control group and the group treated with GW (S1 Fig). The animals were fasted for 12 hours and anaesthetized with intraperitoneal injections of ketamine (Ketamidor®, Richter Pharma AG, Wels, Germany) (100 mg/kg) and xylazine (Xilagesic® 2%, Calier, Barcelona, Spain) (7.5 mg/kg). The mice were then administered DNBS (2.5 mg dissolved in 100 μl of 50% ethanol) using a 4-cm intracolonic catheter (n° 6) [25]. They were maintained in a downright position until recovery from anaesthesia. The body weight, incidence of diarrhoea, and water and food consumption were monitored daily throughout the experiment.

Disease development was assessed using the Disease Activity Index (DAI), which considers three parameters: weight loss, stool consistency and either blood in the perianal region or occult blood in the stool (Feca-cult ®Inlab kit). The animals were anesthetized and euthanized by cervical dislocation on the 17th day of the experiment (S1 Fig).

The colon was then removed aseptically and washed with saline solution (0.9%), and the weight and length (from the ileocecal junction to the anal margin) were measured to calculate the weight/length (W/L) ratio.

### Explants

Once the colon was opened longitudinally, 3 intestinal explants (3 mm of diameter) were obtained and incubated at 37°C in 1 mL of Roswell Park Memorial Institute (RPMI)-1640 culture medium containing 4.5 g/L glucose supplemented with 10% (v/v) foetal bovine serum, 1% penicillin/streptomycin, 1% amphotericin and 2% glutamine in a 5% CO_2_ atmosphere.

The explants were cultured overnight and then collected and the medium was transferred to Eppendorf tubes and centrifuged at 4000 g for 10 min at 4°C and frozen until cytokine measurements. The supernatants were collected and kept at -80°C until levels of the cytokines IL-6 and TNF-α were determined by Enzyme-Linked Immunosorbent Assay (ELISA) using kits from R&D Systems (Minneapolis, MN, USA) following the manufacturer’s protocols. The results are expressed as the concentration of cytokine (pg/mL).

### Analysis of gene expression in colon samples by RT-qPCR

The colon samples were stored in RNAlater^®^ for total RNA isolation. TRIzol^®^ was used for RNA extraction following the manufacturer's instructions. All of the RNA samples were quantified with a NanoDrop^TM^ 2000 spectrophotometer (Thermo Fisher Scientific Inc., Waltham, MA, USA), and 2 μg of RNA was reverse transcribed using oligo(dT) primers (Promega, Madison, WI, USA). Real-time quantitative PCR amplification and detection were performed in optical-grade 48-well plates in an Eco^TM^ PCR Real-Time Optical System (Illumina, San Diego, CA, USA) using 20 ng of cDNA, KAPA SYBRs FAST qPCR Master Mix (Kapa Biosystems, Wilmington, MA, USA) and specific primers at a concentration of 10 μM (S1 Table).

The thermal cycling program consisted of DNA polymerase activation for 2 min at 95°C, followed by 40 reaction cycles as follows: denaturation, 8 s (95°C); annealing, 20 s (at the particular annealing temperature (Ta) for each pair of primers); and extension, 5 s (80°C). Fluorescence was measured at the end of the annealing period of each cycle to monitor the progress of amplification, and a dissociation curve (or melt curve) was added to confirm the amplification specificity of the signal for each case. The gene expression of glyceraldehyde 3-phosphate dehydrogenase (GAPDH) was measured and used to normalize the mRNA expression. The relative RNA levels were calculated using the ΔΔCT method (a comparison of the Ct values of the sample gene and normalizing gene)—ΔΔCT = ΔCTsample- ΔCTreference.

### Histopathological evaluation

Colon samples were collected and fixed in buffered paraformaldehyde (10% in PBS, pH 7.2) for 24 hours. Cross sections were selected and embedded in paraffin. Tissue sections (5 μm, n = 5) were taken and stained with haematoxylin and eosin for histological evaluation by optical microscopy. The criteria for determining the microscopic damage (degree of leukocyte infiltration and the presence/absence of indicators of the inflammatory process) were evaluated by a pathologist [26].

### Myeloperoxidase (MPO) activity

MPO activity was measured according to the technique described by Krawisz et al. [27], and the results were expressed as MPO units per gram of wet tissue; one unit of MPO activity was defined as that degrading 1 mmol hydrogen peroxide/min at 25°C.

### Immunohistochemical analysis of iNOS, p38 MAPK, NF- κB p65 and SOCs-1

Thin colon sections (3 μm, n = 5) were taken, transferred to silanized slides (Dako, Glostrup, Denmark) and subjected to deparaffinization and hydration processes. The intestinal tissue was then washed with 0.3% Triton X-100 in phosphate buffer, treated with 3% hydrogen peroxide, and incubated overnight at 4°C with the following primary antibodies: iNOS, 1:500, p38 MAPK, 1:400, NF-κB p65, 1:100 and SOCS-1, 1:800 (Santa Cruz Biotechnology, Interprise, Brazil). After the slices were washed with phosphate buffer, they were incubated with a streptavidin-HRP-conjugated secondary antibody (Biocare Medical, Concord, CA, USA) for 30 min. Immunoreactivity was visualised with a colourimetric-based detection kit following the protocol provided by the manufacturer (TrekAvidin-HRP Label + Kit from Biocare Medical, Dako, USA) [28].Known positive and negative controls were included in each batch using planimetry microscopy (Olympus BX50, Morphology Department/ UFRN) with a high-powered lens (40x). Immunostaining intensity was determined, and the following scores from 1 to 4 were given: 1, absence of positive cells; 2, small number of positive cells or isolated cells; 3, moderate number of positive cells; 4, large number of positive cells. Labelling intensity was evaluated by two previously-trained examiners in a double-blind fashion. Three sections were evaluated per animal.

### Confocal immunofluorescence analysis

Three tissue sections (n = 5) were deparaffinised with xylene and washed with various concentrations of ethanol and PBS. Antigen retrieval was performed with 10 mM sodium citrate and 0.05% Tween 20 for 40 min at 95°C, while 0.1% Sudan black in 70% ethanol for 40 min at room temperature was used to reduce the autofluorescent background. Sections were incubated overnight with primary antibodies (IL-17, 1:400 and ZO-1, 1:100, Santa Cruz Biotechnology, Interprise, Brazil) and then washed three times in PBS/0.2% Triton X-100 for 5 min and incubated with Alexa Fluor 488-conjugated goat anti-rabbit secondary antibody (1:700 in 1% BSA) and DAPI (Sigma Chemicals) [29].

### In vitro studies

The murine Raw 264 macrophages and CMT-93 rectal carcinoma cell lines were obtained from the Cell Culture Unit of the University of Granada (Granada, Spain). Cells were cultured at 37°C in high glucose (4.5 g/L) modified RPMI-1640 supplemented as described before in a 5% CO_2_ atmosphere. Both cell lines were sub-cultured and used after exponential growth.

Cells were seeded onto 96-well plates and incubated with various concentrations of GW (0.1, 1.0, 10, and 100 μg/mL). After 2 h, Raw 264 and CMT-93 cells were stimulated with lipopolysaccharides (LPS) from *Escherichia coli* O55:B5 (100 ng/mL and 10 μg/mL, respectively) for 24 and 72 h, respectively.

Supernatants from Raw 264 cells were collected after 24 h, and nitrite levels were measured by the Griess reaction (1% sulphanilamide, w/v, in 5% phosphoric acid and 0.1% N-1-naphthyl-ethylenediamine, w/v, in water) [30]. The photometric absorbance at 550 nm was determined to assess nitrite concentration [31]. CMT-93 supernatants were collected after 72 h of stimulation, and IL-6 levels were evaluated by ELISA.

### Statistical analysis

The results are expressed as the mean ± SEM. Differences between the means were tested using one-way analysis of variance (ANOVA) and Tukey’s test. Analyses were performed using GraphPad 6.0 (GraphPad Software Inc., La Jolla, CA, USA), and statistical significance was set at P < 0.05.

## Results

### Chemical characterization of goat whey

The chemical analysis of GW including protein, total lipid, fatty acid, lactose and oligosaccharide contents is presented in Table 1. Among the fatty acids found in GW, there was 1.92 g.100g^-1^ of saturated fatty acids (22.21% stearic acid—C18: 0), 0.63 g.100g^-1^ of monounsaturated fatty acids (vaccenic C18: 1n7 and oleic Z C18: 1n9) and 0.01 g.100g^-1^ of poly-unsaturated fatty acids (Z linoleic C18: 2n6) from total lipids (2.56 ± 0.16 g.100g^-1^).

**Table 1:** Composition of goat whey (GW).

Components	Goat Whey
Protein (g. 100g^-1^)	5.96 ± 0.30
Total lipids (g. 100g^-1^)	2.56 ± 0.25
Fatty acids (g. 100g^-1^)	
Undecylic acid (C11:0)	-
Lauric acid (C12:0)	0.02
Tridecylic acid (C13:0)	-
Myristic acid (C14:0)	0.11
Myristoleic acid (C14:1)	-
Pentadecylic acid (C15:0)	0.01
Palmitic acid (C16:0)	1.20
Palmitoleic acid (Z C16:1n9)	0.00
Margaric acid (C17:0)	0.01
Cis-10-Heptadecenoic (C17:1)	-
Stearic acid (C18:0)	0.57
Elaidic acid (C18:1n9)	-
Oleic acid (C18:1n9)	0.32
Vaccenic acid (C18:1n7)	0.31
Linoleic acid (C18:2n6)	0.01
Arachidic acid (C20:0)	-
Linolenic (C18:3n3)	-
Heneicosylic acid (C21:0)	0.00
Lactose (g. 100g^-1^)	15,62 ± 1.08
Oligosaccharides	
Acids (mg/L)	66.63 ± 2.34
N-Acetylneuraminic acid (mg/L)	24.91 ±1.54
N-Glycolylneuraminic acid (mg/L)	41.72 ± 0.80

### Effects of goat whey on intestinal inflammation

**Fig 1:**
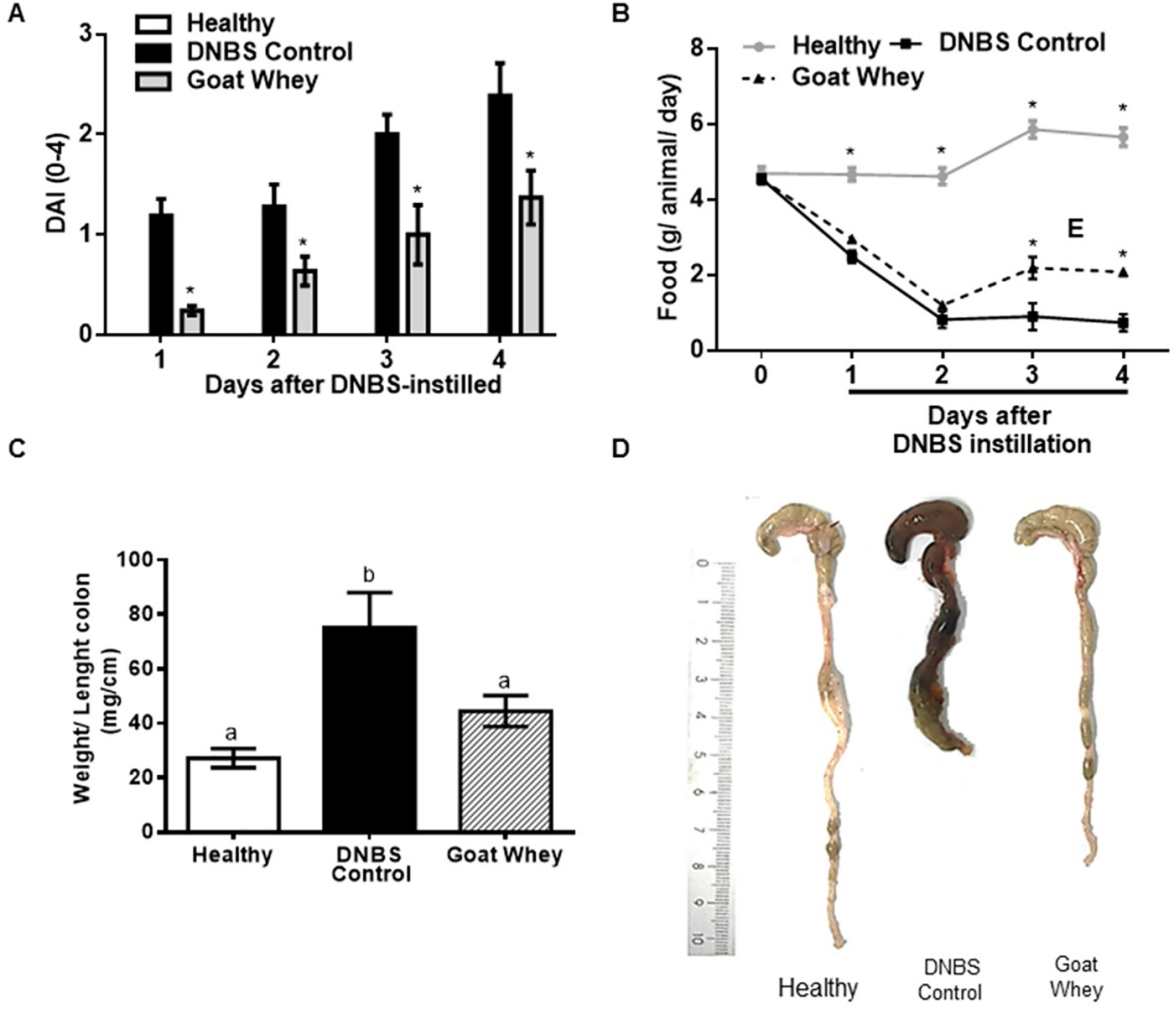
Effects of goat whey on the experimental model of colitis induced by 2,4-dinitrobenzene sulfonic acid (DNBS). (A) Disease Activity Index (DAI); (B) food consumption; (C) weight/length ratio of the colon; and (D) colonic segment of the experimental groups. Data are expressed as the mean ± SEM (n = 12/group). Groups with different letters or with an asterisk (✶) differ significantly (one-way ANOVA post hoc Tukey’s test, P < 0.05).

Treatment with GW had protective effects on the intestinal inflammation induced by DNBS in mice. Rectal administration of DNBS triggered the development of an intestinal inflammatory process that was characterized by weight loss, changes in stool consistency and blood in the perianal region observed on the DNBS control group which had higher DAI values (P < 0.05) than the treated and healthy groups. The mice treated with GW experienced a recovery of the DAI (P < 0.05), on the other hand the DAI of the healthy group was scored as zero (0) and does not appear in the graph bars (Fig 1A).

The W/L ratio of the colon is considered to be an important parameter for the evaluation of intestinal inflammation. The shortening and thickening of the walls of the colonic tissue are common symptoms of intestinal inflammation, and we observed higher values for W/L in the DNBS control group than in the other groups (P < 0.05) (Fig 1C). The colonic damage can be seen in Fig 1D, where a shortening of the colon and the presence of faeces with a less solid consistency and a darker colour were observed when compared with the treated and healthy groups.

The intra-rectal administration of DNBS promoted an intestinal inflammatory process that was characterized by an altered immune response. Various pro-inflammatory cytokines such as IL-1β, IL-17, IL-6 and TNF-α were up-regulated, as well as other key inflammatory players such as iNOS, MMP-9 and ICAM-1, and NF-κB p65 and p38 MAPK signaling pathways. However, the expression of SOCs-1 was down-regulated (P < 0.05) compared with the other groups.

**Fig 2:**
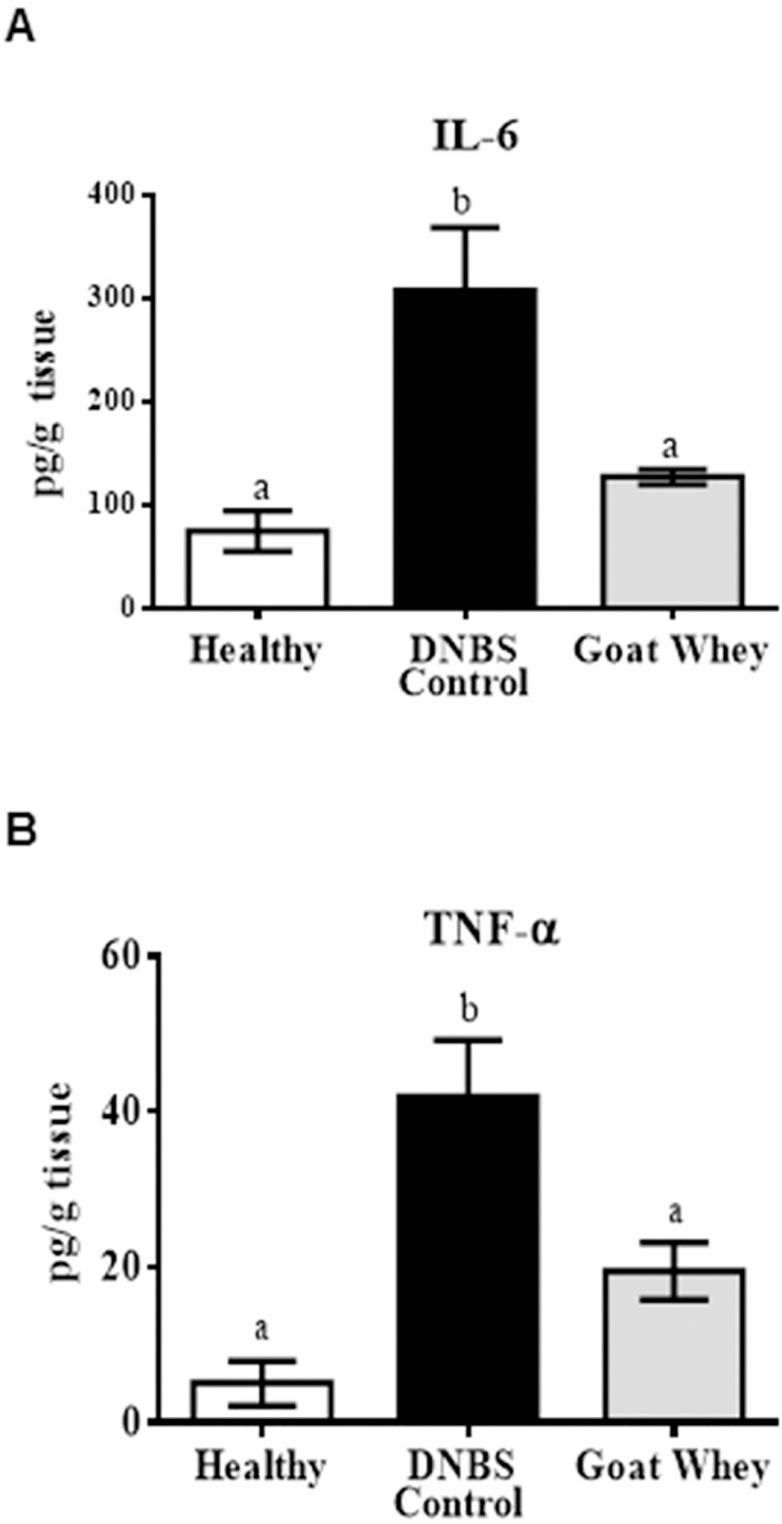
Effects of goat whey on pro-inflammatory cytokines as measured by ELISA. Distal colon tissue samples were cultured overnight. The supernatants were assessed for cytokine levels using kits from R&D Systems (Minneapolis, MN, USA) following the manufacturer’s protocols. The cytokine levels in the supernatant were expressed as the concentration in pg/mL. (A) Interleukin (IL)-6 and (B) tumour necrosis factor (TNF)-α production in colonic tissues from mice with 2,4-dinitrobenzenesulfonic acid (DNBS)-induced colitis. Data are expressed as the mean ± SEM (n = 12). The groups with different letters are significantly different (one-way ANOVA post hoc Tukey’s test, P < 0.05).

GW treatment improved the inflammatory status of the colon, as shown by the assessment of these parameters. Colonic explants from the GW-treated mice produced significantly lower levels of the pro-inflammatory cytokines IL-6 and TNF-α in comparison with the DNBS control group (P < 0.05) (Fig 2).

**Fig 3:**
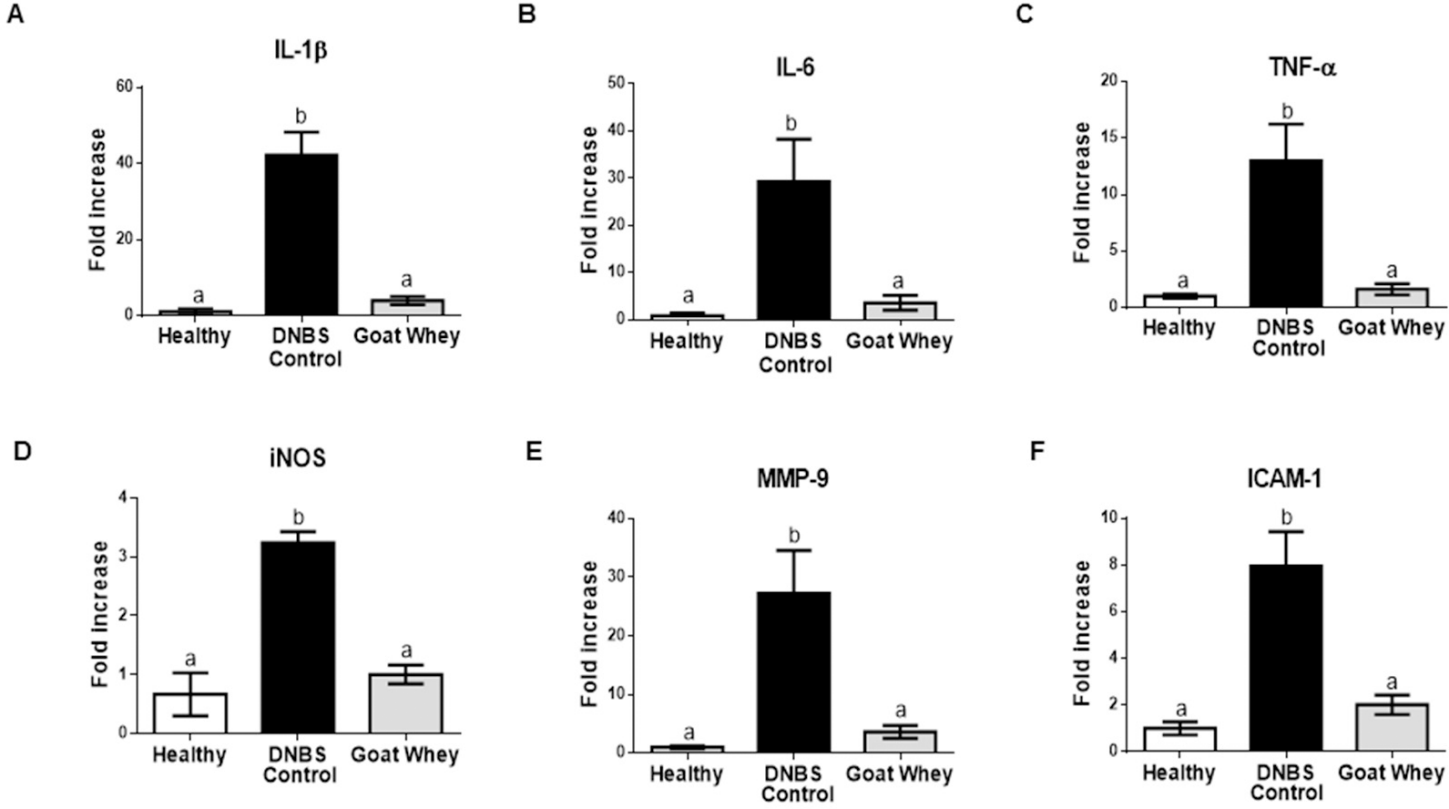
Effects of goat whey on the gene expression of pro-inflammatory cytokines as measured by RT-qPCR. Colonic gene expression of the pro-inflammatory cytokines (A) Interleukin (IL)-1β, (B) IL-6, (C) tumour necrosis factor (TNF)-α, (D) inducible nitric oxide synthase (iNOS), (E) matrix metalloproteinase (MMP)-9, and (F) intercellular adhesion molecule (ICAM)-1 analyzed by real-time qPCR and normalized with the housekeeping gene, Glyceraldehyde-3-phosphate dehydrohenase (GAPDH) in dinitrobenzene-sulphonic acid (DNBS) mice colitis 4 days after damage induction. Data are expressed as the mean ± SEM (n = 12/group). The groups with different letters are significantly different (one-way ANOVA post hoc Tukey’s test, P < 0.05).

Furthermore, the gene expression of all of the pro-inflammatory markers assayed was significantly down-regulated by the treatment (P < 0.05 vs. DNBS control), and the values were similar to those of the healthy group (P > 0.05) (Fig 3 and S2 Fig).

**Fig 4:**
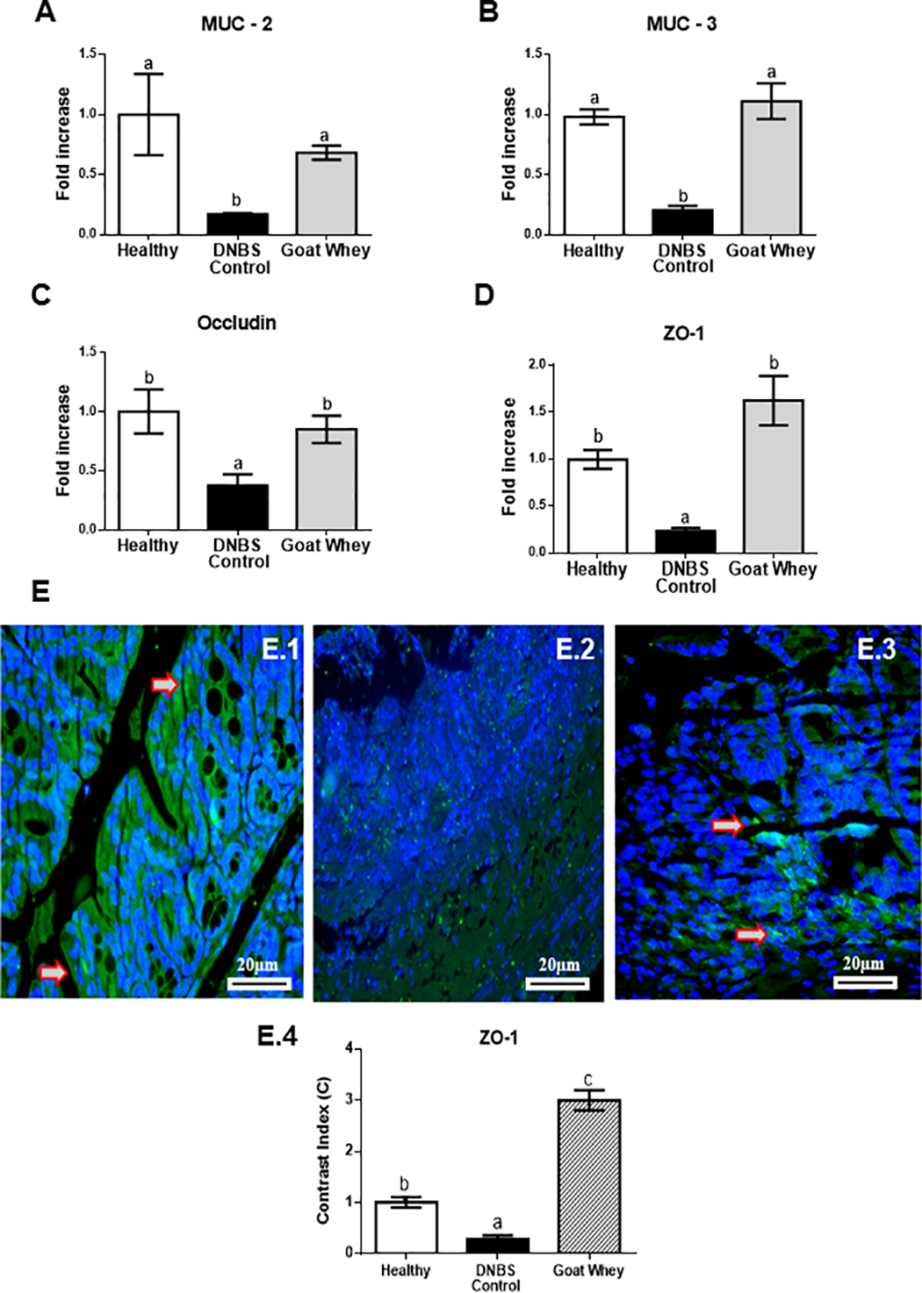
Effects of goat whey on gene expression by RT-qPCR and immunofluorescence of the intestinal mucosal barrier proteins as measured. Colonic gene expression of the barrier function mediators gene expression (A) Mucin (MUC)-2, (B) MUC-3, (C) occludin, (D) zonula occludens (ZO)-1 analyzed by real-time qPCR and normalized with the housekeeping gene, Glyceraldehyde-3-phosphate dehydrohenase (GAPDH) in dinitrobenzene-sulphonic acid (DNBS) mice colitis 4 days after damage induction. Representative confocal photomicrographs of ZO-1 (E) immunoreactivity (green) in colons of the animals from each group; the sections are nuclear counterstained with DAPI (blue): (E.1) Healthy group had moderated ZO-1 labelling; (E.2) ZO-1 labelling was almost absence in DNBS control group; (E.3) ZO-1 labelling (red arrow) was strong in the treated group with goat whey; (E.4) Densitometric analysis confirmed a significant increases in ZO-1 in goat whey. Data are expressed as the means ± SEM. the groups with different letters differ significantly (one-way ANOVA post hoc Tukey’s test, P < 0.05).

DNBS-induced colitis was also characterized by an impairment of intestinal barrier function, as observed by evaluation of the different markers involved in the maintenance of epithelial integrity such as the mucins MUC-2 and MUC-3, occludin, and ZO-1 (Fig 4 and S2 Fig). Treatment with GW also up-regulated the expression of these key proteins compared with the DNBS control group (P < 0.05), which was similar to the healthy group (P > 0.05). Cellular ZO-1 labelling (green) was strong in the GW group (Fig 4E.3), moderated in the healthy group (Fig 4E.1) and almost absent in DNBS control (Fig 4E.2). Densitometric analysis confirmed that there were significantly increased ZO-1 immunoreactivities in GW group (P < 0.05), relative to the DNBS control group. These results showed that an increased expression of ZO-1 corresponds to lower destruction of the intestinal barrier that preserves gut permeability.

**Fig 5:**
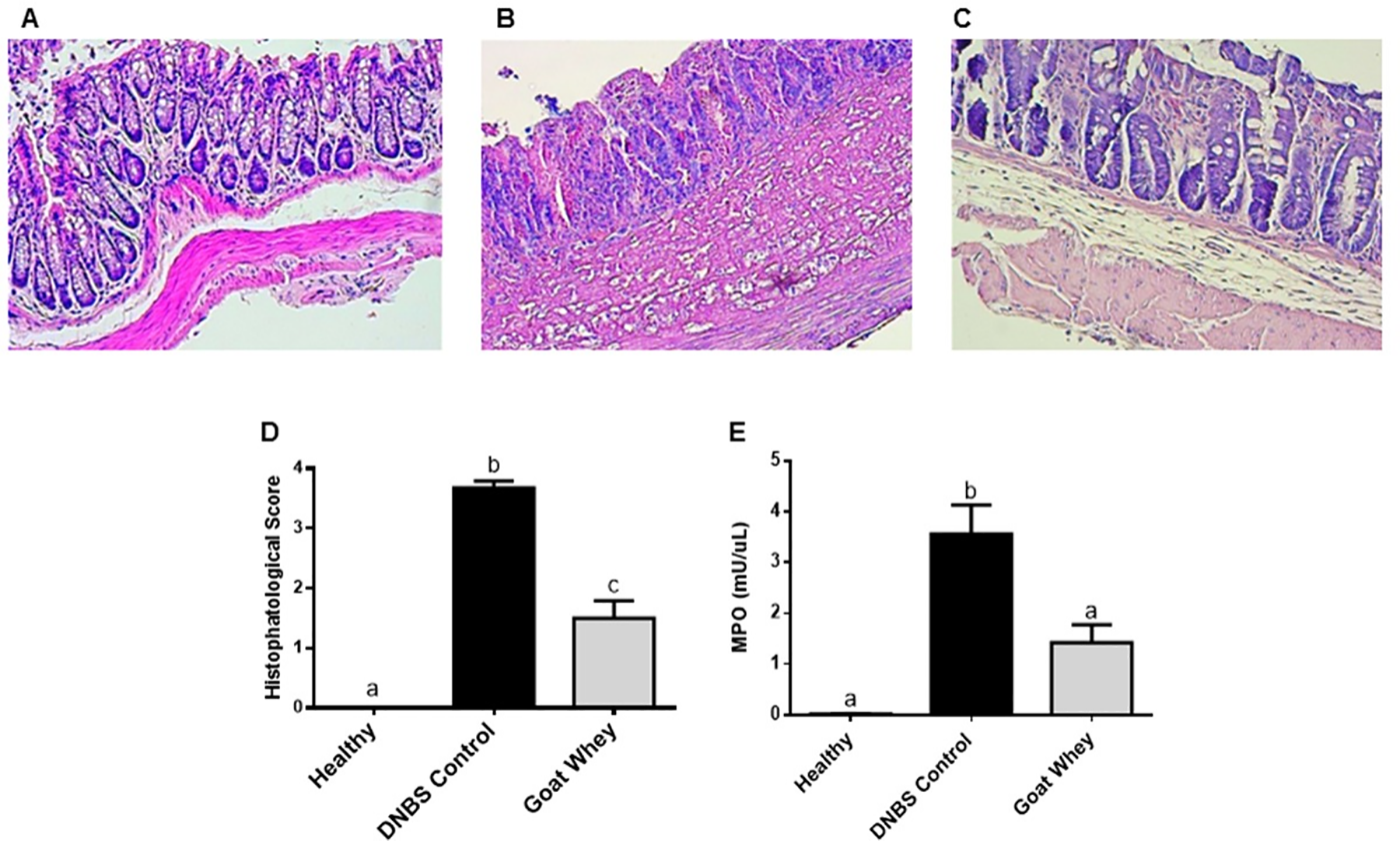
Effects of goat whey on the colonic mucosa of colitic mice as assessed by histological examination. Sections of the colonic mucosa were stained with haematoxylin and eosin (x100): (A) Healthy, (B) DNBS control, and (C) Goat Whey. (D) Microscopic scores were assigned to the different groups according to the criteria described by Zea-Iriarte et al. (1996) [26] and (E) Myeloperoxidase activity–MPO. Data are expressed as the means ± SEM (n = 12/group), and the groups with different letters differ significantly (one-way ANOVA post hoc Tukey’s test, P < 0.05).

Histological assessment of the colon specimens from the DNBS control group showed moderate leukocyte infiltration, a loss of tissue architecture with consequent destruction of the epithelium, a reduction in goblet cells and the presence of haemorrhages (Fig 5B). GW reduced colonic inflammation, thereby preserving the mucosal histology and decreasing neutrophil infiltration (P < 0.05 vs. DNBS control group) (Fig 5C). The colons of the healthy group appeared normal with full organ preservation and the absence of inflammation (Fig 5A).

A reduction (P < 0.05) of the microscopic score (Fig 5D and S2 Fig) in the GW group was followed by a significant reduction (P < 0.05) of the MPO activity (Fig 5E and S2 Fig) compared to the DNBS control group.

**Fig 6:**
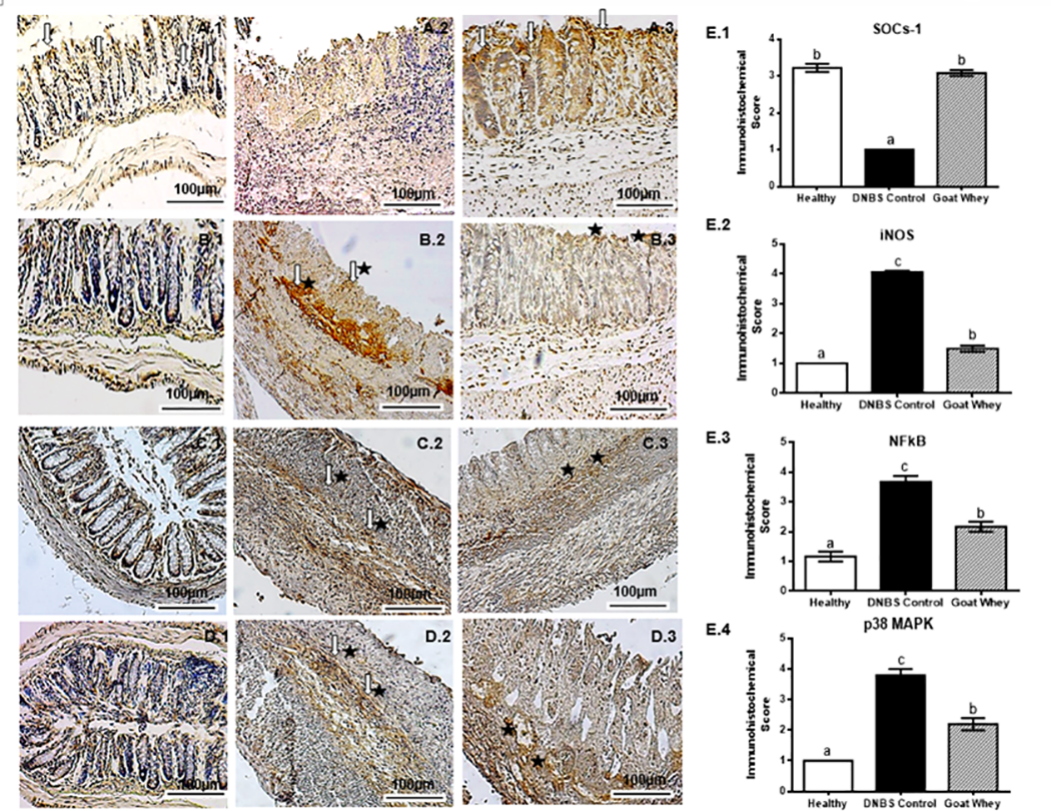
Immunohistochemical analysis of colonic tissue from mice with 2,4-dinitrobenzene sulfonic acid (DNBS)-induced colitis. Effects of goat whey on suppressor of cytokine signalling-1 (SOCs-1) (Panel A), inducible nitric oxide synthase (iNOS) (Panel B), NF-kappaB (NF-κB) p65 (Panel C) and p38 Mitogen Activated Protein Kinases (MAPK) (Panel D). For each antigen, three immunostained sections were examined per animal (n = 5, 3 sections per animal). 40 x magnification, scale bar = 100 μm; 1: Healthy; 2: DNBS Control; 3: Goat Whey; Arrow [⇩] = moderate marking; arrow and star [⇩★] = strong marking; star [★] = weak to moderate marking. Panel E (Immunohistochemical score)—E.1: SOCs-1; E.2: iNOS; E.3: NF-κB p65; E.4: p38 MAPK. Data are expressed as the means ± SEM; the groups with different letters differ significantly (one-way ANOVA post hoc Tukey’s test, P < 0.05).

The results of our immunohistochemical evaluation of the colonic sections were in agreement with the previous results because they showed that DNBS up-regulated the expression of the pro-inflammatory mediator iNOS, which was reduced after the treatment. Moreover, the levels of the inflammatory modulator SOCs-1 were diminished in the DNBS control group and normalized in the GW group (P < 0.05) (Fig 6 and S2 Fig).

NF-κB p65 and p38 MAPK are important signaling pathways in experimental and human colitis. Immunohistochemical staining showed inhibition of these pathways in the anti-inflammatory effect of GW on colitis.

As shown in Fig 6C.2 and 6D.2, DNBS activated the expression NF-κB p65 and p38 MAPK (P < 0.05), respectively, in the colonic tissue, compared to the healthy group (Fig 6C.1 and 6D.1). However, treatment with GW significantly reversed this effect (Fig 6C.3 and 6D.3), represented by a marked reduction in the expression of NF-κB and MAPK 38 (P < 0.05) in relation to the DNBS control group. The immunohistochemistry scores can be visualized in Fig 6E.3 and 6E.4. These results corroborate data from the proinflammatory cytokines IL-1β, IL-6 and TNF-α.

**Fig 7:**
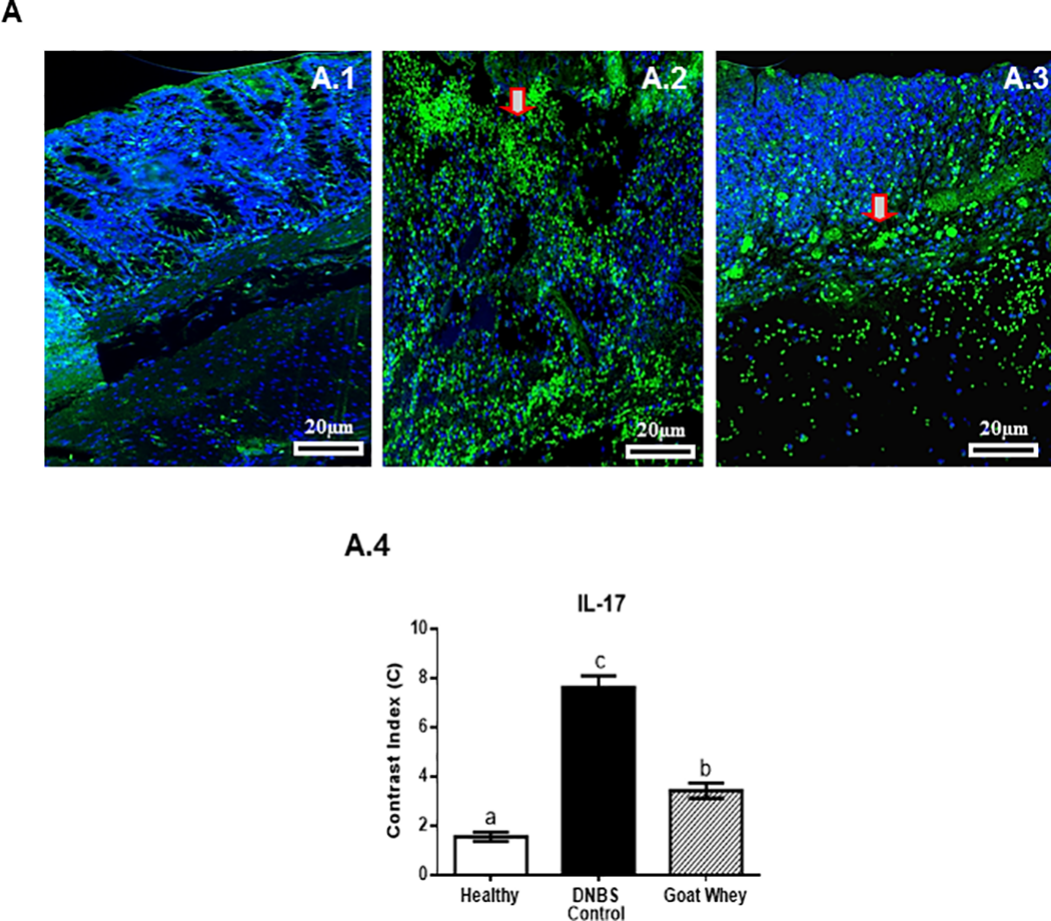
Effect of goat whey on IL-17 expression in colitic mice. Representative confocal photomicrographs of IL-17 (Panel A) immunoreactivity (green) in colons of the animals from each group; the sections are nuclear counterstained with DAPI (blue): (A.1) Healthy group had absent or weak IL-17 labelling in all mucosa layers; (A.2) IL-17 labelling was strong in the DNBS control group; (A.3) weak to moderate IL-17 labelling (red arrow) was seen in the group treated with goat whey; (A.4) Densitometric analysis confirmed a significant reduction in IL-17 immunoreactivity in goat whey. Data are expressed as the means ± SEM; the groups with different letters differ significantly (one-way ANOVA post hoc Tukey’s test, P < 0.05).

The IL-17 signal was strongly diffused (green) in the cells of the DNBS-control group (Fig 7A.2), weak to moderately diffused (green) in all mucosal layers of the GW group (Fig 7A.3), and weak to absent (green) in the healthy group (Fig 7A.1). An increase in the labelling of DAPI showing a decrease in the labelling of IL-17 was observed in the treated and healthy groups. Densitometric analysis confirmed a significant reduction of IL-17 immunoreactivity in the GW group (P < 0.05) (Fig 7A.4 and S2 Fig).

### Effects of goat whey on cellular responses

To characterize the anti-inflammatory effects of the GW components observed in the animal studies, in vitro studies were also conducted using two cell types that are involved in the immune response, Raw 264 macrophages and intestinal epithelial CMT-93 cells.

**Fig 8:**
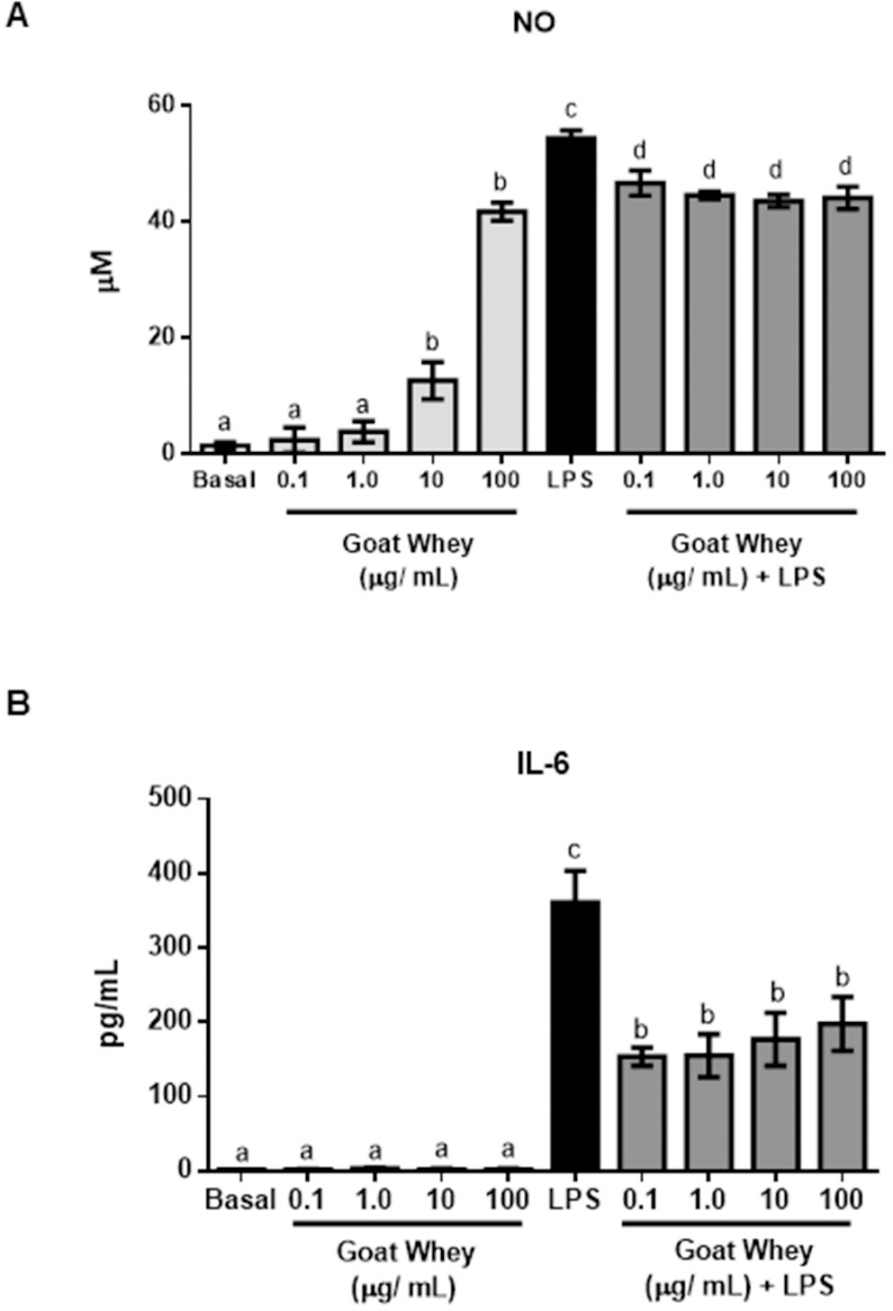
Effects of goat whey on cell lines. (A) Nitrite (NO) production and (B) interleukin (IL)-6 levels in Raw 264 and CMT-93 cells, respectively, in basal or LPS-stimulated conditions (100 ng/mL and 1 μg/mL, respectively). Data are expressed as the mean ± SEM. The bars with different letters are significantly different (one-way ANOVA post hoc Tukey’s test, P < 0.05).

Although GW stimulated NO production in Raw 264 cells at higher concentrations (10 and 100 μg/mL) (P < 0.05 vs. unstimulated untreated cells), after LPS stimulation, GW significantly reduced NO production (P < 0.05 vs. untreated LPS-stimulated cells) (Fig 8A).

In CMT-93 cells, GW had no effect on IL-6 production (P > 0.05 vs. untreated unstimulated cells), but when the cells were stimulated with LPS, GW reduced the production of this cytokine at all concentrations assayed (P < 0.05 vs. untreated LPS-stimulated cells) (Fig 8B). GW did not affect cell viability at any of the concentrations assayed (data not shown).

## Discussion

The results of this study show that GW has preventive effects on DNBS-induced intestinal inflammation in mice, revealing its potential as a functional food that could be useful for the management of human IBD.

Assis et al. (2016) [21] reported a significant improvement in intestinal inflammation in a rat model of acetic acid-induced experimental colitis after treatment with goat milk and goat-derived probiotic yogurt, as evidenced by reductions in macroscopic damage, inflammatory infiltration and oxidative stress, as well as improvements in the cytoarchitecture of the colon.GW improved the clinical signs related to DNBS-induced damage that were evaluated by the DAI. In this regard, GW reduced diarrhoea, weight loss and the presence of blood in the perianal region or occult blood in the stool, as well as the intestinal macroscopic damage and weight/length ratio. The anti-inflammatory activity of GW could be attributed, at least in part, to the presence of polyunsaturated fatty acids and isomers of CLA, such as vaccenic acid and oligosaccharides. Linoleic acid plays an important role in the composition of fatty acids in milk. After the action of the Δ-9 desaturase enzyme, it forms oleic acid, vaccenic acid and rumenic acid, which are also referred to as conjugated linolenic acids (CLAs) [32]. In total, 60 to 95% of the CLA found in milk fat is derived from the action of this enzyme [33]. Interestingly, a recent study reported that feeding with CLA-enriched milk fat, 2% (wt/vol), was effective in reducing the levels of the pro-inflammatory markers of experimental chronic colitis in a mouse model of dextran sulphate-induced colitis [34].

Oligosaccharides are glycans that are composed of lactose with branches of N-acetyllactosamine with sialic acid and fucose on their chains. Sialic acid reduces the adhesion of leukocytes to endothelial cells, indicating that human milk oligosaccharides may have immuno-regulatory effects [35,36]. In addition, oligosaccharides have the ability to stimulate the growth of bifidobacteria in the gastrointestinal tract and beneficial intestinal microbiota in the colon, as well as stimulate the immune system and participate in the defence against bacterial and viral infections by acting as competitive inhibitors of binding sites on the intestinal surface [37,38].

In milk from Saanen and Garganica goats, the levels of sialyl oligosaccharide 3-sialyllactose, 6-sialyllactose and disialyllactose were decreased during days 0–90 of lactation. For Saanen goats, the levels of 3-sialyllactose varied from 176.2 to 106.1 mg/L, while the levels of disialyllactose varied from 146.9 to 27.4 mg/L (Claps et al., 2016). Thum et al. (2015) [39] showed that milk and whey obtained from Saanen goats in New Zealand had high concentrations of sialyl-oligosaccharides (0.20 g/L), suggesting that it may be a useful source to explore the potential health benefits in trials with human. Despite the lower amounts of oligosaccharides, especially sialic acid, in goat milk compared with human milk, they are present at considerable amounts when compared to other ruminants and could be considered as a potential substitute [40,41].

In this study, goat whey reduced the levels of pro-inflammatory cytokines (IL-1β, IL-6 and TNF-α), which could be attributable to the presence of CLA and oligosaccharides. The reduction of IL-6 and TNF-α levels by treatment with GW was also confirmed in ex vivo assays using colonic explants. The elevated expression of IL-1β, IL-6 and TNF-α plays a key role in the pathogenesis of human IBD and experimental colitis [4].

According to previous reports, the expression of proinflammatory cytokines IL-1, IL-6 and TNF-α is mediated by intracellular signal transduction involving the NF-κB pathway and the activation of MAPKs (p38, ERK and JNK) [6]. Once activated, NF-kB also regulates cell proliferation and survival as well as the expression of adhesion molecules (i.e., ICAM) and growth factors, which impact the length and duration of intestinal inflammation. Thus, according to the results obtained we can infer that GW exerts intestinal anti-inflammatory activity through the inhibition of the NF-kB p65/ p38 MAPK signaling pathway.

IL-6 is best described as a pro-tumorigenic cytokine, and together with other members of this family, it affects cell proliferation, survival, differentiation and migration [42], which includes an important role in the pathogenesis of IBD in human and murine by Th1; therefore, reduced IL-6 production is considered to be useful in treating colitis [43,44].

Moreover, inhibition of IL-6 in patients with IBD not only reduces intestinal inflammation but also decreases the risk of developing colorectal cancer [45]. GW induced an inhibitory effect on IL-6 secretion in LPS-stimulated CMT-93 cells. We emphasize that there was no positive regulation of IL-6 production from CMT-93 cells. This finding is supported by the in vivo experiments in which the improvement of murine colitis was also associated with a down-regulation of IL-6 gene expression in colonic tissue.

The mechanisms of modulation of the gene expression of inflammatory proteins include down-regulation of the signalling cytokines and STAT receptors such as the endogenous protein SOCs [46,47]. In this regard, GW treatment promoted an increase in SOCs-1, which may be associated with the reduction in the levels of pro-inflammatory cytokines.

GW also caused a reduction in ICAM-1 levels that may be a result of the improvement in intestinal inflammation. In the intestinal inflammatory process, cells and macrophages can release large amounts of TNF-α. This can increase the expression of adhesion molecules such as VCAM-1 and ICAM-1 in endothelial cells, thereby significantly increasing the infiltration of leukocytes into the intestinal mucosa. It has been shown that blocking one or more of these adhesion molecules can effectively inhibit inflammation, which makes them interesting targets for the development of new therapies [48].

IL-17 is also an attractive therapeutic target, as it is involved in the development of chronic inflammation associated with many inflammatory and autoimmune disorders when produced in excess. As expected, the IL-17 staining was more pronounced in the confocal analysis of the DNBS control group compared with the other groups. However, at lower concentrations, IL-17 plays a key role in host defence against extracellular bacterial and fungal infections [49].

The beneficial effects of GW were also evidenced in the histopathological evaluation of the colonic segments and in the reduction of MPO activity, an important marker of neutrophil infiltration. We observed an improvement in the tissue architecture and an increase in the number of goblet cells, which are important components of the intestinal epithelium that are responsible for producing key peptides for defence and epithelial repair of the intestinal mucosa.

The presence of goblet cells in colonic tissue from animals treated with GW corroborates the above data concerning MUC-2 and MUC-3 regulation. Goblet cells synthesize glycoprotein-secreting mucins (MUC-2) and mucins that are linked to the epithelial membrane (MUC-1, MUC-3), thus protecting the underlying epithelial cells from mechanical damage and the direct actions of ingested chemicals by maintaining the integrity of the intestinal barrier [50–52].

The intestinal epithelium is formed by a cell monolayer that acts as a barrier and is connected with sophisticated cellular junctions, among them occludin and ZO-1 [53]. The up-regulation of these proteins is related to improvements an intestinal permeability [54] and epithelial integrity, as it prevents the bacterial translocation of input antigens and subsequently reduces colitis [55]. GW also promoted a reduction in MMP-9, a family of proteolytic zinc enzymes and calcium-dependent structural proteins that degrade the extracellular matrix and are implicated in the pathogenesis of human IBD and experimental colitis [56].

In addition, iNOS has also been shown to be involved in the pathogenesis of bowel inflammation because an increase in iNOS expression in areas of inflammation has been shown to be associated with histological inflammatory parameters [57]. It has been proposed that the increased amounts of NO produced by iNOS can react with superoxide to form peroxynitrite, which induces deleterious changes in the structure and function of proteins [58]. Thus, the reduction in iNOS gene expression in the group treated with GW may be associated with improvements in the inflamed areas of the colons of these mice. Moreover, the in vitro studies performed in Raw 264 cells, both under basal conditions and after stimulation with LPS (thus simulating an inflammatory environment), were aimed at assessing whether the anti-inflammatory activity of GW was related to inhibition of iNOS enzyme.

The fact that pretreatment of these cells with the highest concentrations of GW resulted in an increase in NO production may indicate that GW activates constitutive nitric oxide synthase (cNOS). In this case, the production of large amounts of NO may be important for protecting against cellular invaders and cell tumours, as well as having beneficial effects on vascular lesions with endothelial cell loss [59]. However, GW decreased nitric oxide production in cells stimulated with LPS, and LPS can directly interact with the apical surface to induce responses in intestinal epithelial cells, which in turn induce the production of cytokines and other inflammation mediators [43].

## Conclusion

GW has revealed itself as a promising candidate for the treatment of IBD. It was able to mitigate the evaluated clinical signs and inhibit the secretion of pro-inflammatory cytokines such as IL-1β, IL-6, IL-17 and TNF-α, through the inhibition of the p38 MAPK/NF-kB p65 signalling pathways, as well as the reduction of iNOS, MMP-9 and ICAM-1, in particular by altering the proprieties of CLA and sialic acid. Moreover, GW increased the expression of the mucins MUC-2 and MUC-3, as well as occludin, ZO-1 and SOCs-1, thus inhibiting the intestinal inflammatory process induced by DNBS. A reduction in inflammation was also evidenced by a decrease in the microscopic damage score of the colonic tissue from the GW-treated group. GW also modulated the effects of iNOS in vitro by reducing nitrite production in Raw 264 cells that were stimulated with LPS, as well as IL-6 production in CMT-93 cells.

## Supporting information

S1 Fig Experimental design. (DOCX)

S2 Fig Individual data used in the experiments. (DOCX)

S1 Table Primer sequences used in real-time qPCR assays involving samples from the model of experimental colitis induced by DNBS. (DOCX)
