# Supplementary figures and images for: Intestinal anti-inflammatory effects of goat whey on DNBS-induced colitis in mice
Source: PLoS One. 2017 Sep 28;12(9):e0185382. doi: 10.1371/journal.pone.0185382 (PMC5619769; doi:10.1371/journal.pone.0185382)

**S1 Fig. Experimental design.**


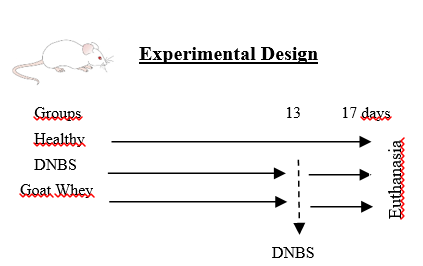


Healthy

DNBS Control

Goat Whey

**Groups**

Euthanasia

13 17 days

DNBS

Supplement: S1 Fig — (DOCX) [file pone.0185382.s001.docx]
