# Supplementary material for: Intestinal anti-inflammatory effects of goat whey on DNBS-induced colitis in mice
Source: PLoS One. 2017 Sep 28;12(9):e0185382. doi: 10.1371/journal.pone.0185382 (PMC5619769; doi:10.1371/journal.pone.0185382)
Supplement: S2 Fig — (DOCX) [file pone.0185382.s002.docx]

**Individual data used in the experiments**

**RT-qPCR (FIG. 3)**

**RT-qPCR (FIG. 4)**

**HISTOPATOLOGICAL SCORE AND MPO (FIG.5)**

**IMMONOHISTOCHEMICAL SCORE (Fig. 6)**

**immunoflurescence (fig. 7)**
