# Supplementary material for: Intestinal anti-inflammatory effects of goat whey on DNBS-induced colitis in mice
Source: PLoS One. 2017 Sep 28;12(9):e0185382. doi: 10.1371/journal.pone.0185382 (PMC5619769; doi:10.1371/journal.pone.0185382)
Supplement: S1 Table — (DOCX) [file pone.0185382.s003.docx]

**S1 Table.** Primer sequences used in real-time qPCR assays, in the model of experimental colitis induced by DNBS.

| Gene |  | Sequence 5′–3′ | | Annealing temperature  (°C) | |
| --- | --- | --- | --- | --- | --- |
| GAPDH | FW | CCATCACCATCTTCCAGGAG | 60 | |  |
|  | RV | CCTGCTTCACCACCTTCTTG |  | |  |
| IL-1β | FW | TGATGAGAATGACCTCTTCT | 55 | |  |
|  | RV | CTTCTTCAAAGATGAAGGAAA |  | |  |
| IL-6 | FW | TAGTCCTTCCTACCCCAATTTCC | 60 | |  |
|  | RV | TTGGTCCTTAGCCACTCCTTC |  | |  |
| iNOS | FW | GTTGAAGACTGAGACTCTGG | 56 | |  |
|  | RV | GACTAGGCTACTCCGTGGA |  | |  |
| ICAM-1 | FW | GAGGAGGTGAATGTATAAGTTATG | 60 | |  |
|  | RV | GGATGTGGAGGAGCAGAG |  | |  |
| MMP-9 | FW | TGGGGGGCAACTCGGC | 60 | |  |
|  | RV | GGAATGATCTAAGCCCAG |  | |  |
| MUC-2 | FW | GATAGGTGGCAGACAGGAGA | 60 | |  |
|  | RV | GCTGACGAGTGGTTGGTGAATG |  | |  |
| MUC-3 | FW | CGTGGTCAACTGCGAGAATGG | 60 | |  |
|  | RV | CGGCTCTATCTCTACGCTCTC |  | |  |
| TNF- α | FW | AACTAGTGGTGCCAGCCGAT | 56 | |  |
|  | RV | CTTCACAGAGCAATGACTCC |  | |  |
| OCLN | FW | ACGGACCCTGACCACTATGA | 56 | |  |
|  | RV | TCAGCAGCAGCCATGTACTC |  | |  |
| ZO-1 | FW | GGGGCCTACACTGATCAAGA | 56 | |  |
|  | RV | TGGAGATGAGGCTTCTGCTT |  | |  |

Legend: GAPDH: Glyceraldehyde-3-phosphate dehydrogenase; IL-1β: Interleukin-1β; IL-6: Interleukin-6; iNOS: Inducible nitric oxide synthase; ICAM-1; Intercellular adhesion molecule-1; MMP-9: Metalloproteinase -9; MUC-2: Mucin-2; MUC-3: Mucin-3; OCLN: Occludin; TNF- α: Tumour necrosis factor- α; ZO-1: Zonula occludens-1.
